# Accelerometer-derived “weekend warrior” and regular physical activity on long-term risk of irritable bowel syndrome and subsequent depression: a large-scale prospective cohort study

**DOI:** 10.3389/fpubh.2026.1865206

**Published:** 2026-06-30

**Authors:** Chujie Tu, Xin Yang, Yan Wang, Jiaqi Hu, Xiaohua Hou, Tao Bai, Li Liu

**Affiliations:** 1 Division of Gastroenterology, Tongji Medical College, Union Hospital, Huazhong University of Science and Technology, Wuhan, Hubei, China; 2 Department of Endoscopy, Zhejiang Cancer Hospital, Hangzhou Institute of Medicine (HIM), Chinese Academy of Sciences, Hangzhou, Zhejiang, China; 3 Ministry of Education Key Lab of Environment and Health, Department of Epidemiology and Biostatistics, School of Public Health, Tongji Medical College, Huazhong University of Science and Technology, Wuhan, Hubei, China; 4 Department of Gastroenterology, College of Medicine, Tianyou Hospital, Wuhan University of Science and Technology, Wuhan, China; 5 Department of Clinical Medicine, College of Medicine, Wuhan University of Science and Technology, Wuhan, China; 6 Division of Gastroenterology, Tongji Medical College, The Central Hospital of Wuhan, Huazhong University of Science and Technology, Wuhan, China; 7 Hubei Provincial Clinical Research Center for Colorectal Cancer, Wuhan, Hubei, China; 8 Wuhan Clinical Research Center for Colorectal Cancer, Wuhan, Hubei, China

**Keywords:** accelerometer, depression, irritable bowel syndrome, physical activity, weekend warrior

## Abstract

**Background:**

The WHO recommends ≥150 min/week of moderate-to-vigorous physical activity (MVPA) for optimal health. Whether weekend warrior (WW) or regular physical activity (PA) patterns differently influence risks of irritable bowel syndrome (IBS) and subsequent depression is unclear. This study explored these associations using accelerometer-derived PA.

**Methods:**

We included 84,799 UK Biobank participants with one-week accelerometer data to assess incident IBS using Cox models. Restricted cubic splines evaluated dose–response relationships and identified optimal and minimal candidate MVPA doses. Participants were classified as inactive, WW, or regularly active using guideline (150 min/week) and statistical thresholds. Among 3,976 baseline IBS patients, guideline-based groups were used to examine MVPA and subsequent depression risk with Cox models.

**Results:**

Over a median follow-up of 8.0 years, 962 participants developed IBS. MVPA showed a nonlinear inverse association, plateauing at 225 min/week, with a minimal candidate dose of 80 min/week. Both WW and regular activity were linked to lower IBS risk using 150 min/week (WW: HR 0.75, 95% CI 0.65–0.87; regular: HR 0.73, 95% CI 0.61–0.88) and 80 min/week thresholds (WW: HR 0.75, 95% CI 0.64–0.88; regular: HR 0.68, 95% CI 0.56–0.83). Among IBS patients, both WW (HR 0.61, 95% CI 0.42–0.89) and regular activity (HR 0.65, 95% CI 0.41–1.03) demonstrated comparable inverse associations with subsequent depression, with no significant difference between the two patterns.

**Conclusion:**

Both WW and regular MVPA were similarly associated with a lower IBS risk, with minimal and optimal candidate doses of 80 and 225 min/week, respectively. Among IBS patients, both activity patterns demonstrated comparable inverse associations with subsequent depression, although findings were constrained by limited statistical power.

## Introduction

Irritable bowel syndrome (IBS) is a prevalent disorder of gut-brain interaction characterized by recurrent abdominal pain accompanied by altered bowel habits (1, 2). Globally, IBS affects approximately 5–10% of the population, imposing substantial economic burdens on healthcare systems (3, 4). Due to persistent symptoms, IBS patients often experience comorbid psychiatric disorders, especially depression (5, 6). Given the limitations of current targeted therapies, identifying modifiable lifestyle factors is critical for reducing the risk of IBS (7).

Physical activity (PA) is widely recognized for its beneficial effects on gastrointestinal health and is recommended by the British Society of Gastroenterology as a first-line intervention for IBS management (8). The World Health Organization (WHO) advises adults to engage in 150–300 min of moderate-to-vigorous physical activity (MVPA) per week to achieve substantial health benefits, with additional benefits beyond 300 min (9). However, existing guidelines do not specify optimal MVPA patterns, and predominantly rely on self-reported questionnaire data, which often correlate weakly with objectively measured accelerometer data (10, 11). To date, a dearth of attention has been paid to investigating the association between the accelerometer-measured PA duration and the incidence of IBS. Most previous studies were limited by small sample sizes (12), or reliance on self-reported activity (13). In addition, the WHO Guidelines Development Group has highlighted the need for large-scale prospective observational studies employing device-based measures to objectively elucidate the dose–response relationships for various health outcomes (14).

Due to contemporary lifestyle constraints, regular weekday exercise is often challenging for many individuals. Some individuals, termed “weekend warrior (WW)”, opt to concentrate their weekly MVPA into 1 or 2 days (15). This condensed PA pattern might offer a more feasible and practical approach for risk reduction in the general population, particularly considering the higher morbidity of IBS among younger adults (16). Besides, the prevalence of depression is notably elevated among IBS patients (5, 6), significantly impairing their quality of life and exacerbating the overall disease burdens. Additionally, mental healthcare visits constitute a larger proportion of direct medical costs in IBS patients compared to gastroenterology visits (17). PA, being relatively easier to maintain and more cost-effective than prescription medications (18), holds potential for lowering the risk of subsequent depression. However, it remains unclear how PA duration and patterns may be associated with depression among IBS patients.

To bridge these research gaps, we leveraged accelerometer-derived data from the UK Biobank to investigate the associations between PA duration and patterns with incident IBS risk. Additionally, we conducted secondary analyses to further explore their relationship with depression among patients with baseline IBS.

## Materials and methods

### Study design and participants

The UK Biobank is a large-scale cohort comprising approximately half a million participants aged 37–73 years, recruited between 2006 and 2010 across 22 assessment centers in England, Wales, and Scotland, as previously described (19). At baseline, participants completed comprehensive questionnaires on sociodemographic, lifestyle, and health-related information, underwent physical examinations, and provided biological samples. Ethical approval was granted by the North West Multi-centre Research Ethics Committee (reference: 11/NW/0382). Written informed consent was obtained from all participants. The present analysis was conducted under UK Biobank application number 88159.

**Figure 1:**
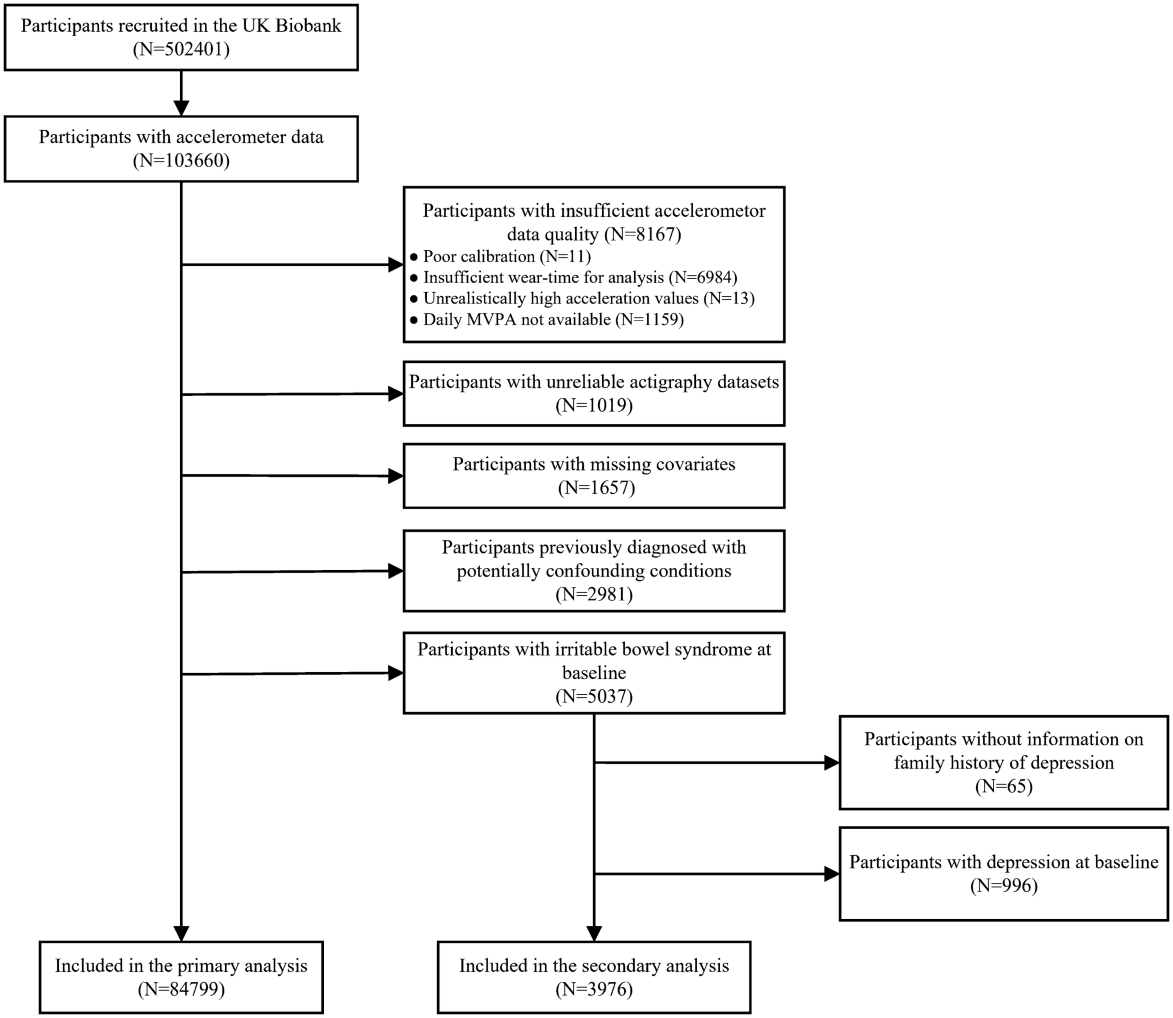
Flowchart of participant inclusion for the primary and secondary analyses.

**Figure 2:**
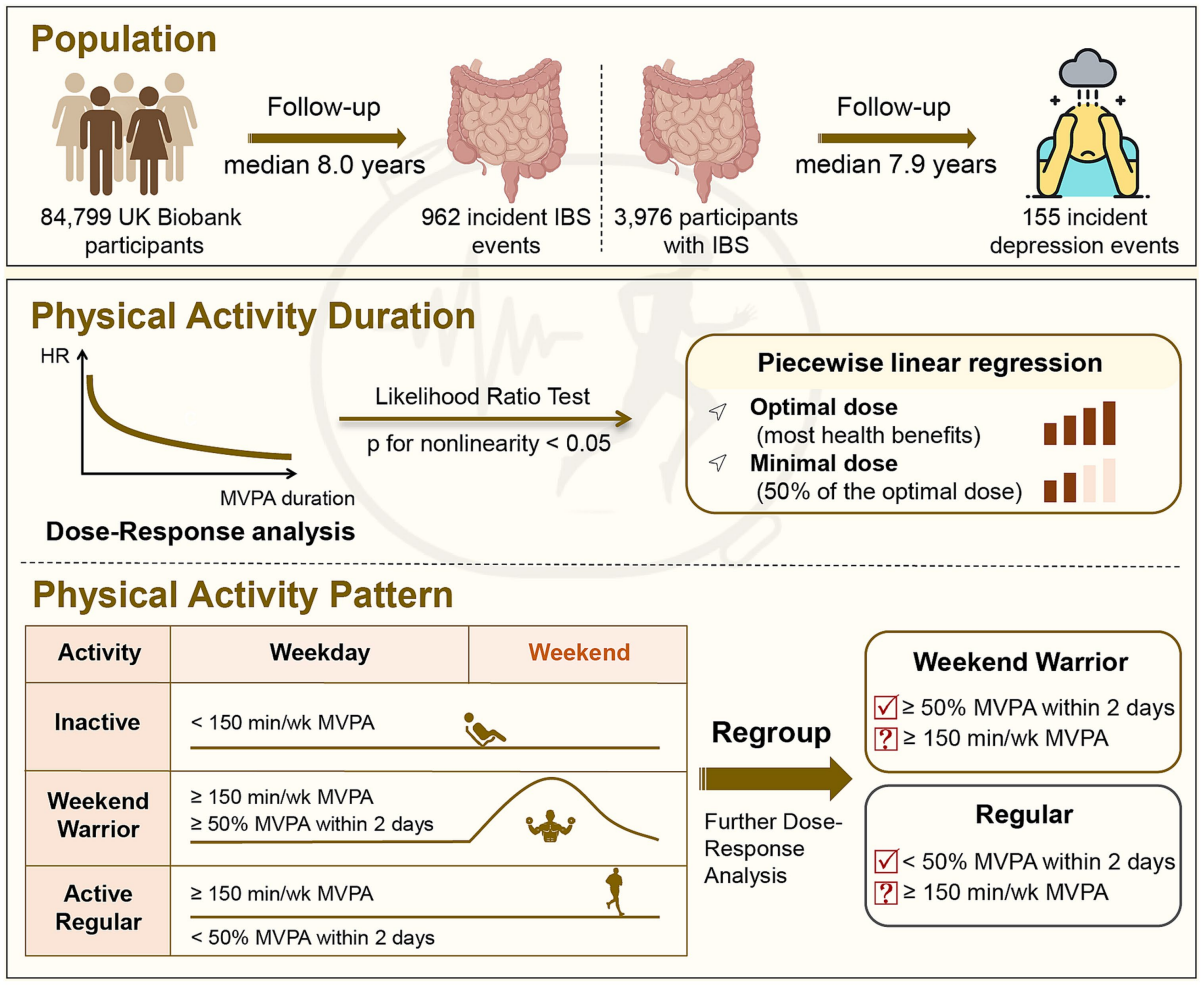
Study overview. From 84,799 UK Biobank participants with valid accelerometry data, 962 incident IBS cases were identified over a median follow-up of 8.0 years. Among 3,976 participants with baseline IBS, 155 developed subsequent depression. Physical activity was assessed by both duration and patterns. Dose–response relationships were examined by restricted cubic splines. When nonlinearity was detected (*p* < 0.05), two-piecewise linear regression was used to identify optimal and minimal candidate doses. Participants were classified as: inactive (<150 min/week MVPA), weekend warriors (≥150 min/week MVPA with ≥50% concentrated within 1–2 days), or active regular (≥150 min/week MVPA but not weekend warriors). MVPA, moderate-to-vigorous physical activity; IBS, irritable bowel syndrome. Created with BioRender.com.

In this study, we initially included 103,660 participants with available accelerometer data. Exclusions were made for insufficient accelerometer data quality (*N* = 8,167), unreliable actigraphy data (*N* = 1,019), incomplete covariate information (*N* = 1,657), prior diagnosis of potential confounding diseases (*N* = 2,981), including inflammatory bowel disease (IBD; ICD-10 K50-K51), gastrointestinal malignancy (C15-C26), malabsorption syndromes (K90), and baseline IBS (*N* = 5,037). Conversely, for the secondary analysis investigating subsequent depression, we specifically utilized these 5,037 participants with baseline IBS. From this group, we further excluded those lacking information on family history of depression (*N* = 65) and those with depression at baseline (*N* = 996). Ultimately, 84,799 participants were included in the primary analysis, with 3,976 participants included in the secondary analysis (Figure 1). The study design is summarized in Figure 2.

### Exposure assessment

Participants were instructed to wear Axivity AX3 triaxial accelerometers on their dominant wrist continuously for 1 week (20). Acceleration data were recorded at 100 Hz with a dynamic range of ±8 g and aggregated into 5-s epochs (mean vector magnitude). Detailed procedures for accelerometer data collection and processing have been described previously (15, 21). MVPA was distinguished from sedentary behavior, light physical activity, and sleep using a validated machine learning algorithm (22).

Given the lack of optimal threshold for MVPA measured by wrist-worn accelerometers (23), in the main analyses, we assessed the guideline-based threshold. Participants were classified into 3 groups: (1) active WW, achieving ≥150 min/week MVPA with ≥50% concentrated over any 1–2 days of the week (not necessarily weekend days) (15); (2) active regular, meeting ≥150 min/week MVPA but not qualifying as active WW; and (3) inactive, accumulating <150 min/week MVPA. Further, we tested additional thresholds defined by dose–response analysis.

### Study outcome assessment

This study focused on the incident IBS and depression in IBS patients. The UK Biobank captures the first occurrence dates of health outcomes mapped to three-character International Classification of Disease (ICD) codes sourced from primary care data, hospital inpatient data, death register records and self-reported medical conditions. IBS (ICD-10: K58) and depression (ICD-10: F32 and F33) diagnoses were thus identified accordingly. Follow-up extended from the date participants completed accelerometry until the date of the earliest outcome diagnosis, death, lost to follow-up, or the end of follow-up, whichever came first.

### Covariates assessment

Potential confounders were preliminarily selected based on prior literature (15, 24). In line with previous UK Biobank accelerometry studies (15, 25), the date of accelerometer wear (June 2013–December 2015) served as the analytical baseline for all time-to-event analyses. Covariates were predominantly ascertained at the original UK Biobank assessment visit (2006–2010) through standardized questionnaires and physical examinations. These included age, sex, race, smoking status, body mass index (BMI), alcohol grams, educational attainment, diet quality, employment status, and Townsend deprivation index (TDI). However, approximately 10% of participants in our cohort attended the first repeat assessment visit (August 2012–June 2013), which occurred in closer temporal proximity to accelerometer wear. For these individuals, we systematically updated their covariates using data from the repeat visit to minimize any potential exposure–covariate time mismatch. In secondary analysis exploring depression outcomes, family history of depression was further included. Details on covariate assessments are provided in the eMethods of Supplementary material.

### Statistical analysis

Baseline characteristics were summarized by incident IBS (yes or no), with mean (SD) for continuous variables and percentages for categorical variables. Differences between groups were assessed by Wilcoxon rank-sum tests for continuous variables and chi-square tests for categorical variables. Additionally, to evaluate potential selection bias, standardized mean differences (SMDs) were calculated to compare baseline characteristics between the overall UK Biobank cohort and the accelerometer sub-cohort, as well as between participants included in and excluded from the primary analysis (with an SMD > 0.10 indicating a meaningful difference) (26). Because the proportion of participants excluded due to missing covariate data was extremely low (~1.6%), a complete-case approach was adopted, as multiple imputation is unlikely to meaningfully alter the estimates when missingness is under 5%.

Cox proportional hazard regression models were employed to estimate the hazard ratios and 95% confidence intervals (CIs) for the association of PA duration and patterns with outcomes. The proportional hazards assumption was tested via Schoenfeld residuals, and no violations were detected. Model 1 was adjusted for age (continuous) and sex, and Model 2 was further adjusted for race (white or non-white), smoking status (never, former or current), alcohol grams (continuous), educational attainment (continuous), BMI (<25.0, 25.0–29.9, ≥30.0), Townsend deprivation index (continuous), employment status (yes or no), diet quality (good, normal or poor). Family history of depression was additionally adjusted in the analysis of the association between PA and depression in IBS patients.

Dose–response relationships between PA duration and outcomes were assessed using fully-adjusted restricted cubic spline (RCS) regression. For IBS, four knots were positioned at the 5th, 35th, 65th, and 95th percentiles of PA duration, while three knots (10th, 50th, 90th percentiles) were used for depression outcomes due to the smaller sample size. If non-linearity was detected (*P* for non-linearity < 0.05), a threshold analysis was conducted via two-piecewise linear regression (21). Likelihood ratio tests combined with bootstrap resampling were used to determine the threshold. The optimal candidate PA dose for health benefits was defined as the inflection point of the two-piecewise linear regression, and the minimal candidate dose corresponded to the MVPA duration achieving 50% of the maximal risk reduction observed at the optimal dose based on prior literature (27, 28). Stratified analyses for IBS incidence across different PA duration and patterns were performed to investigate potential subgroup differences.

To assess the robustness of the results, sensitivity analyses were conducted by removing participants with incident outcomes in the previous 2 years after the completion of accelerometer measurement to mitigate the probability of reverse causation, removing subjects diagnosed with conditions resembling IBS symptoms, including noninfective gastroenteritis, colitis, and other pancreatic disorders, or removing BMI from the covariate list due to its possible mediation effect. Furthermore, to address potential residual confounding, we conducted an additional sensitivity analysis by further adjusting for sleep duration, sedentary time, baseline gastrointestinal medication use, and overall health status.

A two-tailed *p* < 0.05 was considered to be statistically significant. Analyses were performed using R version 4.3.2.

## Results

### Population characteristics in primary analysis

A total of 84,799 participants were included, with a mean age of 61.81 (SD, 7.86) years, and 46,947 (55.4%) were female (Table 1). As shown in Supplementary Table S1, compared with the overall UK Biobank cohort, the accelerometer sub-cohort exhibited slightly healthier profiles (e.g., lower BMI and a lower proportion of current smokers), reflecting a moderate healthy volunteer effect. However, when comparing participants included in versus excluded from our primary analysis within the accelerometer subset, baseline characteristics were highly comparable, with all SMDs < 0.10 (Supplementary Table S2), indicating that the complete-case approach introduced minimal additional selection bias. During a median follow-up of 8.0 years [interquartile range (IQR), 7.4–8.5 years], 962 participants (1.13%) developed IBS. Compared with those who did not develop IBS, participants with incident IBS were more likely to be female and unemployed, had lower educational attainment, lower alcohol intake, and higher BMI.

**Table 1:** Baseline characteristics of participants in UK Biobank by incident irritable bowel syndrome in the primary analysis.

Characteristic	Incident IBS			*P*-value^2^
	Overall	No	Yes	
	*N* = 84,799^1^	*N* = 83,837^1^	*N* = 962^1^	
Age (years)	61.81 (7.86)	61.80 (7.86)	62.30 (7.58)	0.089
Sex				<0.001
Female	46,947 (55.4)	46,259 (55.2)	688 (71.5)	
Male	37,852 (44.6)	37,578 (44.8)	274 (28.5)	
Race/ethnicity				0.725
Non-white	2,654 (3.1)	2,622 (3.1)	32 (3.3)	
White	82,145 (96.9)	81,215 (96.9)	930 (96.7)	
Body mass index				**0.021**
Normal (<25.0)	33,237 (39.2)	32,874 (39.2)	363 (37.7)	
Overweight (25.0–29.9)	35,059 (41.3)	34,681 (41.4)	378 (39.3)	
Obese (≥30.0)	16,503 (19.5)	16,282 (19.4)	221 (23.0)	
Smoking status				0.338
Current	5,712 (6.7)	5,650 (6.7)	62 (6.4)	
Former	30,520 (36.0)	30,152 (36.0)	368 (38.3)	
Never	48,567 (57.3)	48,035 (57.3)	532 (55.3)	
Alcohol intake (g/week)	129.13 (136.51)	129.41 (136.68)	105.20 (118.78)	**<0.001**
Diet quality				0.068
Good	14,911 (17.6)	14,715 (17.6)	196 (20.4)	
Normal	48,520 (57.2)	47,983 (57.2)	537 (55.8)	
Poor	21,368 (25.2)	21,139 (25.2)	229 (23.8)	
Townsend Deprivation Index	−1.73 (2.82)	−1.73 (2.81)	−1.65 (2.95)	0.840
Employment status				**0.003**
Employed	51,493 (60.7)	50,953 (60.8)	540 (56.1)	
Unemployed	33,306 (39.3)	32,884 (39.2)	422 (43.9)	
Educational attainment (years)	15.39 (4.64)	15.40 (4.64)	14.56 (4.71)	**<0.001**
Physical activity pattern				**<0.001**
Inactive	28,028 (33.1)	27,606 (32.9)	422 (43.9)	
Weekend warrior	36,336 (42.8)	35,986 (42.9)	350 (36.4)	
Active regular	20,435 (24.1)	20,245 (24.1)	190 (19.8)	

^1^Mean (SD) for continuous; *n* (%) for categorical. ^2^Wilcoxon rank sum test; Pearson’s Chi-squared test. Bold values indicate statistical significance (*P* < 0.05).

### Association of PA duration and IBS

**Figure 3:**
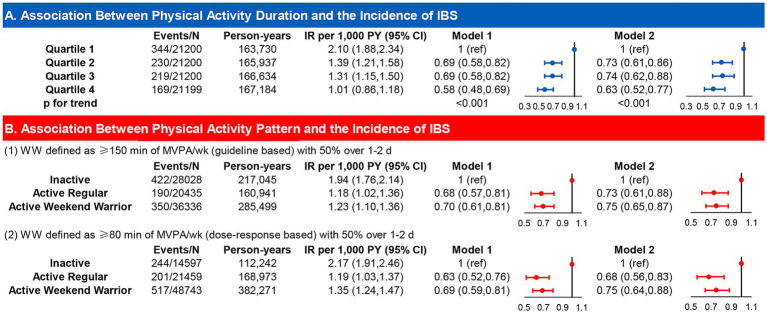
Associations of physical activity duration and patterns with incident IBS. **(A)** Analysis of MVPA duration and IBS incidence. **(B)** Physical activity patterns stratified by (1) guideline-recommended threshold (≥150 min/week MVPA) and (2) minimal candidate dose threshold (≥80 min/week MVPA). Weekend warriors were defined as achieving the specified MVPA threshold with ≥50% concentrated within 1–2 days; active regular participants met the threshold but with <50% within 1–2 days; inactive participants did not meet the threshold. Data are presented as hazard ratios (HRs) with 95% confidence intervals (CIs). Model 1: adjusted for age (continuous) and sex. Model 2: additionally adjusted for race (white or non-white), smoking status (never, former, or current), alcohol intake (grams/week, continuous), educational attainment (years, continuous), BMI (<25.0, 25.0–29.9, or ≥30.0 kg/m^2^), Townsend Deprivation Index (continuous), employment status (employed or unemployed), and diet quality (good, normal, or poor). IBS, irritable bowel syndrome; MVPA, moderate-to-vigorous physical activity; BMI, body mass index; IR, incidence rate; PY, person-years.

Associations between PA duration and IBS incidence were shown in Figure 3. The multivariable-adjusted associations were consistent in both Model 1 (HR _Q4 vs Q1_ = 0.58; 95% CI: 0.48, 0.69) and Model 2 (HR _Q4 vs Q1_ = 0.63; 95% CI: 0.52, 0.77), with both *P* for trend < 0.001 (Figure 3A). These associations were generally consistent across predefined subgroups of age, sex, BMI, alcohol intake, smoking status, diet, educational attainment, employment status and TDI (Supplementary Figure S1), with a significant interaction observed in the educational attainment subgroup (*p* = 0.040).

**Figure 4:**
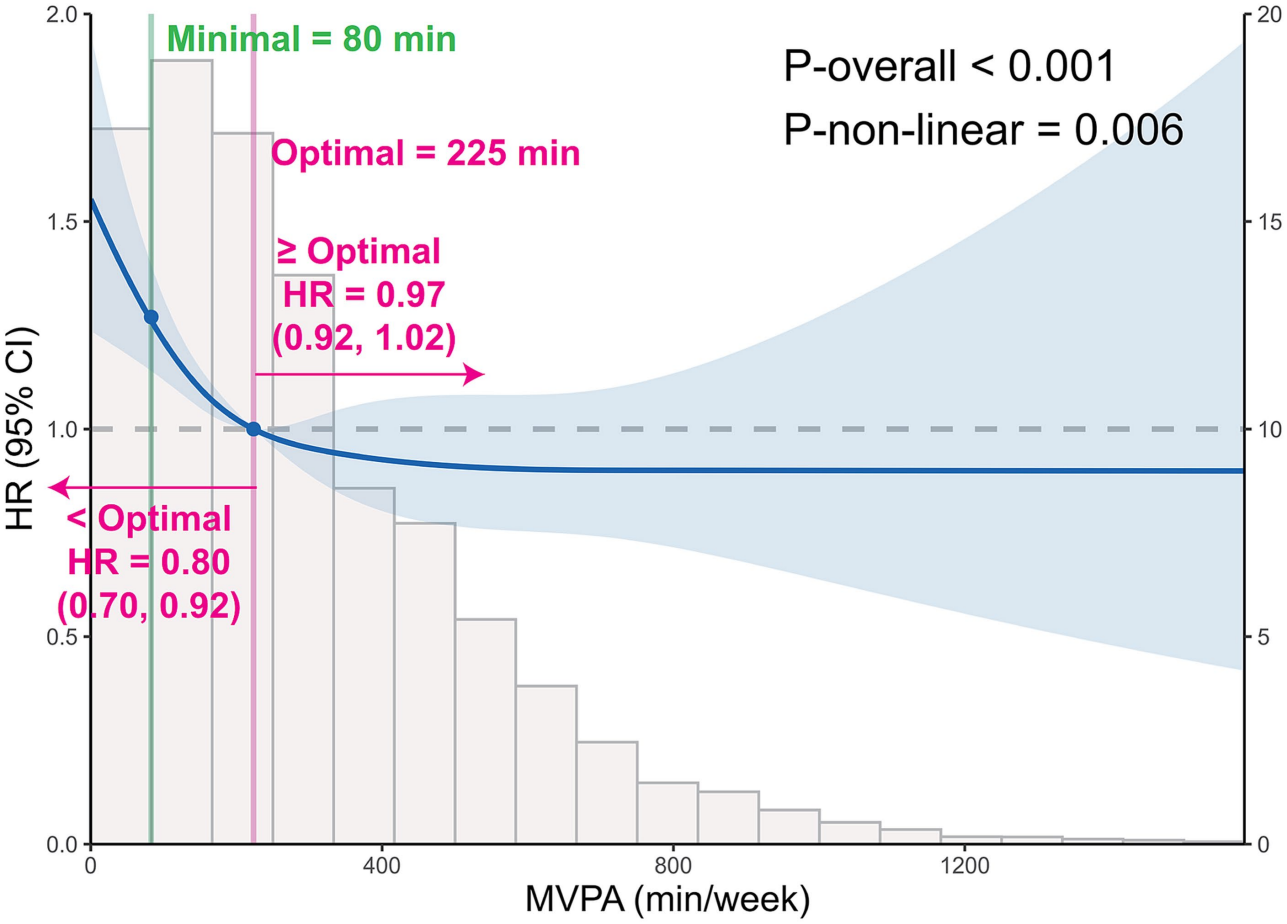
Dose–response analysis of physical activity duration (min/week) with incident IBS risk in the entire study population. Hazard ratios are indicated by solid lines and 95% CIs by shaded areas. Histogram shows the distribution of physical activity duration. Model adjusted for age (continuous), sex, race (white or non-white), smoking status (never, former, or current), alcohol intake (grams/week, continuous), educational attainment (years, continuous), BMI (<25.0, 25.0–29.9, or ≥30.0 kg/m^2^), Townsend Deprivation Index (continuous), employment status (employed or unemployed), and diet quality (good, normal, or poor). Optimal dose: the inflection point where most IBS risk reduction is achieved. Minimal dose: the activity level that provides approximately half the risk reduction of the optimal dose. IBS, irritable bowel syndrome; MVPA, moderate-to-vigorous physical activity; BMI, body mass index.

Subsequently, multivariable-adjusted RCS exhibited an L-shape dose–response relationship between PA duration and incident IBS (*P*-overall < 0.001, *P*-non-linear = 0.006) (Figure 4). Threshold analysis using two-piecewise linear regression identified 225 min/week (95% CI: 220–231 min/week) as the optimal candidate PA dose; IBS risk decreased steeply with increasing MVPA below this threshold (per 15-min/day increment: HR = 0.80; 95% CI: 0.70–0.92) but plateaued thereafter (per 15-min/day increment: HR = 0.97; 95% CI: 0.92–1.02). Based on 50% of the risk reduction observed at the optimal candidate dose, the minimal candidate dose was identified at 80 min/week.

### Association of PA patterns and IBS

According to the guideline-based threshold (150 min/week), participants were classified as active WW (*N* = 36,336, 42.8%), active regular (*N* = 20,435, 24.1%), and inactive (*N* = 28,028, 33.1%) (Figure 3B). Both active WW (HR 0.75, 95% CI 0.65–0.87) and active regular (HR 0.73, 95% CI 0.61–0.88) showed a lower risk of incident IBS compared with inactive participants (Figure 3B). Using the statistical minimal dose (80 min/week), a larger proportion of participants met the active criteria (WW: *N* = 48,743, 57.5%; regular: *N* = 21,459, 25.3%), with similar risk reductions observed for WW (HR 0.75, 95% CI 0.64–0.88) and regular activity (HR 0.68, 95% CI 0.56–0.83) (Figure 3B).

Kaplan–Meier curves for each PA pattern revealed comparable cumulative IBS risk for the active WW and active regular groups (Supplementary Figure S2A). Additionally, similar inverse dose–response associations of MVPA duration and the risk of incident IBS for both WW and regular PA patterns across the entire range of MVPA (Supplementary Figure S3). Stratified analyses demonstrated consistent associations between PA patterns and risk of IBS, with no significant interaction observed in different subgroups (Supplementary Figure S4).

### Association of PA and depression in IBS patients

**Figure 5:**
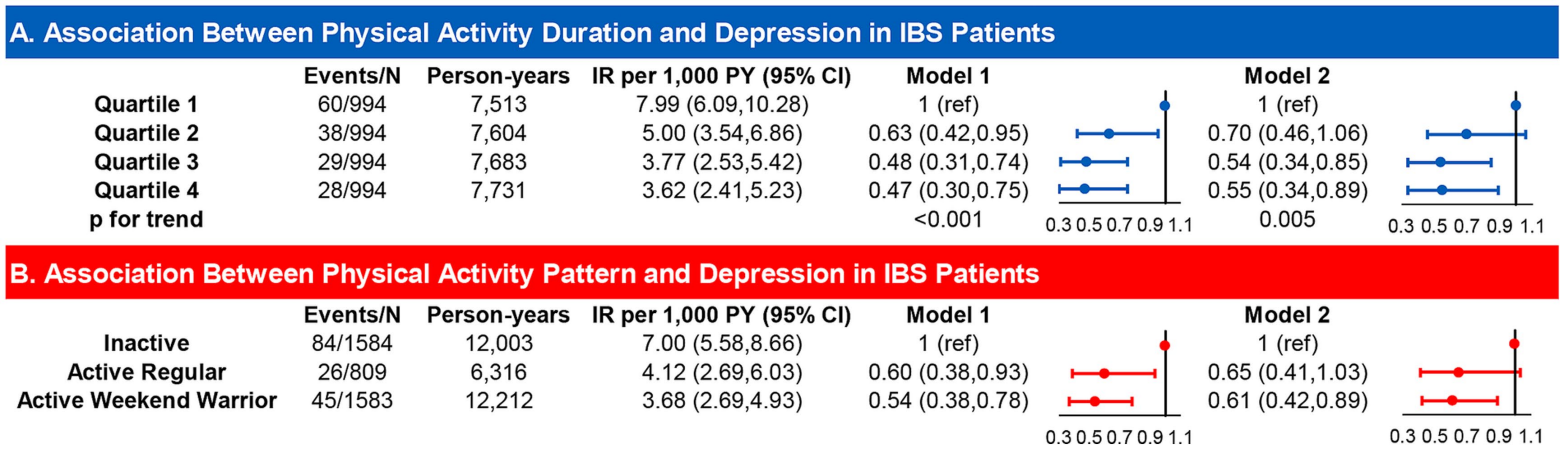
Association of physical activity duration and patterns with subsequent depression risk in IBS patients. **(A)** Analysis of MVPA duration and incident depression among IBS patients. **(B)** Analysis of physical activity patterns and incident depression among IBS patients. Weekend warriors were defined as achieving ≥150 min/week MVPA with ≥50% concentrated within 1–2 days; active regular participants met ≥150 min/week MVPA but with <50% within 1–2 days; inactive participants accumulated <150 min/week MVPA. Data are presented as hazard ratios (HRs) with 95% confidence intervals (CIs). Model 1: adjusted for age (continuous) and sex. Model 2: additionally adjusted for race (white or non-white), smoking status (never, former, or current), alcohol intake (grams/week, continuous), educational attainment (years, continuous), BMI (<25.0, 25.0–29.9, or ≥30.0 kg/m^2^), Townsend Deprivation Index (continuous), employment status (employed or unemployed), diet quality (good, normal, or poor), and family history of depression (yes or no). IBS, irritable bowel syndrome; MVPA, moderate-to-vigorous physical activity; BMI, body mass index; IR, incidence rate; PY, person-years.

**Figure 6:**
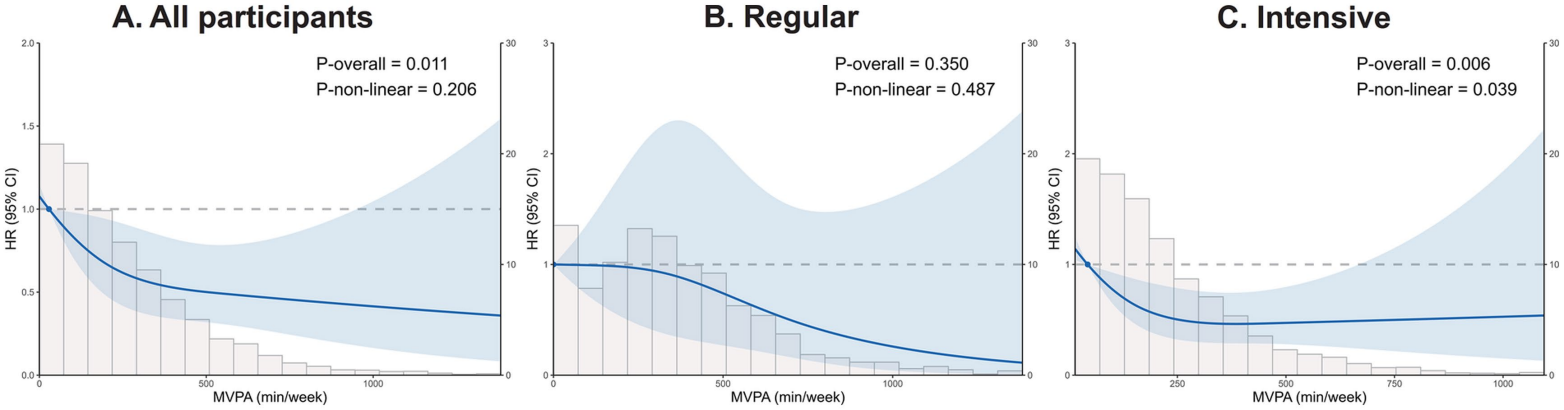
Dose–response analysis of physical activity duration (min/week) with the subsequent depression risk among all IBS patients. **(A)** All participants. **(B)** Regular group. **(C)** Weekend warrior group. Hazard ratios are indicated by solid lines and 95% CIs by shaded areas. Histogram shows the distribution of physical activity duration. Model adjusted for age (continuous), sex, race (white or non-white), smoking status (never, former or current), alcohol grams (continuous), educational attainment (continuous), BMI (<25.0, 25.0–29.9 or ≥30.0 kg/m^2^), Townsend deprivation index (continuous), employment status (yes or no), diet quality (good, normal or poor) and family history of depression (yes or no).

During the follow-up of 7.9 years [interquartile range (IQR), 7.3–8.4 years], 155 (3.9%) incident depression cases were documented among 3,976 IBS patients (Supplementary Table S3). Consistent with the IBS findings, higher PA was significantly associated with reduced depression risk in both Model 1 (HR _Q4 vs Q1_ = 0.47; 95% CI: 0.30, 0.75) and Model 2 (HR _Q4 vs Q1_ = 0.55; 95% CI: 0.34, 0.89) as shown in Figure 5A. However, the dose–response relationship was linear rather than non-linear (*P*-overall = 0.011, *P*-non-linear = 0.206) (Figure 6).

Thus, based on the guideline recommendations, participants were categorized into inactive (*N* = 1,584, 39.8%), active regular (*N* = 809, 20.3%), or active WW (*N* = 1,583, 39.8%) groups. Among IBS patients, multivariable-adjusted models showed a significantly lower risk of subsequent depression in the active WW group (HR = 0.61; 95% CI: 0.42–0.89), whereas the reduction in the active regular group was not statistically significant (HR = 0.65; 95% CI: 0.41–1.03) (Figure 5B). Importantly, a direct comparison between the active WW and active regular groups revealed no significant difference in depression risk (HR = 1.07; 95% CI: 0.66–1.74) (Supplementary Table S4). This indicates that the lack of statistical significance in the regular group is likely attributable to the smaller number of events rather than a difference in the underlying association.

Comparable cumulative depression risk in IBS patients were observed for the active WW and active regular groups (Supplementary Figure S2B). In RCS analysis among the entire range of MVPA, a non-linear association was shown in the WW group (*P*-overall = 0.006, *P*-non-linear = 0.039), but not in the regular group (*P*-non-linear = 0.487) (Figure 6).

### Sensitivity analyses

In the sensitivity analyses, removing participants with incident outcomes in the previous 2 years after accelerometry, removing subjects diagnosed with conditions resembling IBS symptoms, or removing BMI from the covariate list did not substantially change the magnitude of any of the associations observed (Supplementary Figures S5–S9). Importantly, the inverse associations of both the active WW and regularly active patterns against incident IBS and subsequent depression remained robust in the additional sensitivity analysis that further adjusted for sleep duration (Supplementary Table S5), sedentary time (Supplementary Table S6), baseline gastrointestinal medication use (Supplementary Table S7), and overall health status (Supplementary Table S8).

## Discussion

In this study, using data from 84,799 UK Biobank participants with week-long wrist-worn accelerometers, we examined the associations of PA patterns with incident IBS and subsequent depression. MVPA showed a nonlinear, L-shaped association with incident IBS, with candidate minimal and optimal doses of approximately 80 min/week and 225 min/week. Both regularly active and WW participants had similarly lower IBS risk. Among patients with baseline IBS, higher MVPA was associated with lower risk of subsequent depression, with both the WW and regular patterns demonstrating comparable inverse associations. These findings were robust across sensitivity analyses, highlighting that sufficient PA, even if concentrated over 1–2 days, may be a potential approach warranting further investigation for mitigating gastrointestinal and mental health risks.

To our knowledge, several prospective studies have examined associations of accelerometer-measured WW and regular activity patterns with various health outcomes, including cardiovascular disease (29–31), diabetes (32–34), anxiety and depression (35, 36), Parkinson’s disease (37), and brain health (25, 38, 39). These studies consistently indicate that accumulating MVPA in 1–2 days per week or spreading it across multiple days can be associated with similar risk reductions for these outcomes. However, no study has examined the relationship between these activity patterns and IBS using accelerometer-measured PA. Using large-scale accelerometer data, our study is the first to demonstrate that both the WW and regular activity patterns are similarly associated with a lower IBS risk. Potential biological mechanisms may explain why the WW pattern confers similar benefits for gut health as regular PA. Both patterns share common pathways involving gut microbiota modulation, short-chain fatty acid (SCFA) production, gut–brain axis regulation, and systemic inflammation reduction (40–42). Aerobic exercise, whether concentrated on 1–2 days or distributed across the week, increases the diversity and abundance of beneficial bacteria such as Lactobacillus and Bifidobacterium (43, 44). These bacteria ferment dietary fibers into SCFAs (e.g., butyrate, acetate, propionate), which strengthen the intestinal barrier, reduce gut permeability, and exert local anti-inflammatory effects (45, 46). Notably, even short-duration, high-dose MVPA can transiently accelerate gut motility and shorten intestinal transit time, thereby limiting pathogenic overgrowth and promoting a healthier microbial environment. In addition, exercise-induced activation of the gut–brain axis contributes to the observed equivalence. Concentrated MVPA reduces cortisol levels and enhances neurotransmitters such as 5-hydroxytryptamine and *γ*-aminobutyric acid, which regulate mood and visceral perception (47, 48). Given that IBS is a prototypical gut–brain interaction disorder, these neurobiological changes alleviate both gastrointestinal and psychological symptoms, irrespective of exercise distribution (49, 50). Furthermore, both activity patterns lower pro-inflammatory cytokines (e.g., TNF-*α*, IL-6) (51, 52), reducing gut-specific and systemic low-grade inflammation, and restoring immune homeostasis and visceral hypersensitivity (53, 54). Collectively, this mechanistic evidence supports that total weekly MVPA volume—rather than its temporal distribution—is the key factor associated with lower IBS risk (55, 56). Therefore, the WW pattern may yield comparable risk reductions to regular activity through shared biological pathway*s.* This finding suggests that for individuals with busy schedules, WW may offer a practical approach to achieve recommended PA levels and potentially lower IBS risk.

Although activity patterns provide important insights, the total weekly amount of PA required to manage or lower the risk of IBS remains unclear. Multiple randomized controlled trials (RCTs) have demonstrated that in patients with established IBS, 12-week moderate-intensity aerobic exercise interventions, performed 3–5 days per week for 30–60 min per session, significantly improve symptoms and quality of life (57–59). Moreover, prospective studies also suggest that regular PA may be associated with a reduced risk of developing IBS (13). These findings collectively highlight the therapeutic and risk-reducing potential of PA for IBS. However, previous evidence has primarily relied on self-reported PA, resulting in uncertainty regarding the optimal objectively measured weekly activity for IBS risk reduction in the general population. Our study extends this evidence by objectively demonstrating a nonlinear L-shaped association between MVPA and incident IBS. More specifically, IBS risk decreased progressively with increasing MVPA. Consistent with prior dose–response literature, we identified an exploratory minimal candidate dose of approximately 80 min/week corresponding to about half of the maximal risk reduction, and maximal risk reduction observed around 225 min/week, beyond which additional activity was associated with limited further risk reduction. Although WHO guidelines recommend ≥300 min/week of MVPA for broader health benefits, accelerometer-based studies consistently demonstrate diminishing marginal returns beyond a certain threshold (11, 21, 28), and our findings support this pattern. From a public health perspective, increasing activity levels among insufficiently active individuals may be associated with greater reductions in IBS incidence than encouraging already active individuals to further increase their PA.

IBS is a prototypical gut–brain interaction disorder, with patients exhibiting a high burden of anxiety and depression (60–62). Extensive evidence indicates that regular PA confers significant benefits for emotional regulation (63–67), and accelerometer-based studies further demonstrate that maintaining high levels of PA—whether concentrated or distributed—is associated with a lower risk of depression and anxiety (36). However, due to the increased psychological risk in IBS patients, objective data on the association between PA and subsequent depression remain limited. Our study found an inverse association between MVPA duration and depression risk in IBS patients, indicating that even at moderate-to-high activity levels, additional PA may be associated with a further lower risk of depressive symptoms. This finding challenges the commonly held notion of a plateau effect of exercise benefits in the general population (67, 68), implying that effective emotional regulation in IBS may be associated with higher levels of activity. Notably, stratified analyses by activity pattern revealed considerable heterogeneity in these associations. In the WW group, MVPA showed a significant nonlinear association with depression risk, with inverse associations evident even at relatively low activity levels. Both the active WW and regularly active groups demonstrated similar point estimates for risk reduction. Although the association in the regularly active group did not reach statistical significance, our direct comparison between the two patterns showed no significant difference. This underscores that the observed discrepancy in statistical significance is driven primarily by limited statistical power—specifically, the small number of depression events in the active regular group—rather than the WW pattern being superior in its association with psychological protection. Sensitivity analyses further confirmed the robustness of these findings. In summary, our study highlights that the WW pattern represents a flexible and potential practical approach for the psychological management of IBS patients.

In subgroup analyses, we also noted a statistically significant interaction for educational attainment (*p* = 0.040). This may reflect differences in socioeconomic resources, health literacy, healthcare access, or other unmeasured lifestyle factors; however, given multiple subgroup comparisons and the modest *p* value, this result should be interpreted cautiously.

This study has several strengths. First, we leveraged a large cohort of UK adults with long duration of follow-up, enhancing the generalizability of the conclusion. Second, PA was measured via accelerometers, which minimized recall bias and provided more granular information on daily activities. Third, outcomes were identified through multiple data sources, and participants diagnosed with conditions resembling IBS symptoms were excluded, thus improving accuracy in outcome ascertainment. Fourth, we conducted a dose–response analysis of objectively measured PA duration and IBS incidence, identifying a nonlinear relationship and estimating both the optimal and minimal candidate activity doses, thereby offering exploratory evidence to inform future guidelines on physical activity for IBS risk reduction. Finally, comprehensive sensitivity analyses confirmed the robustness and validity of our findings.

Several limitations should also be considered. First, IBS patients were identified using linked health records and administrative ICD-10 codes rather than the clinical gold standard of Rome IV symptom criteria. These administrative codes do not capture IBS subtypes, symptom severity, or symptom duration, which inevitably leads to the underdiagnosis of milder cases and underestimation of the true incidence. However, because MVPA was measured objectively and independently of healthcare-seeking behavior, any outcome misclassification is likely non-differential with respect to exposure, biasing hazard ratios toward the null (i.e., the true magnitude of the inverse association may be stronger than observed). Second, accelerometer data were collected over only 7 days at baseline, which precluded us from accounting for long-term changes in activity patterns during the 8-year follow-up. Although prior studies in the UK Biobank suggest moderate long-term reproducibility of device-measured activity (69), within-person variability and regression dilution remain possible. Because this exposure misclassification is likely non-differential, it would generally attenuate hazard ratios toward the null, suggesting that the observed associations may actually underestimate the true risk reduction associated with usual long-term MVPA. Future studies leveraging repeated PA measurements over longer periods are encouraged to validate these findings. Third, our findings are based on participants aged 37–73 years, limiting generalizability to other age groups, particularly those ≥80 years. Furthermore, participants in the UK Biobank, particularly those in the accelerometer sub-cohort, exhibit a “healthy volunteer effect.” While this may introduce selection bias regarding absolute incidence rates, prior methodological studies demonstrate that exposure-disease associations remain widely generalizable despite such selection mechanisms. Fourth, while we adjusted for a validated overall diet quality index, the UK Biobank baseline questionnaire lacks the granularity to capture specific dietary components crucial to IBS pathogenesis, such as detailed dietary fiber or FODMAP intake. Consequently, residual confounding by these specific dietary factors cannot be entirely ruled out. Fifth, our secondary analysis investigating incident depression among IBS patients was inherently underpowered. Because only 155 depression events occurred among the 3,976 baseline IBS patients, the confidence intervals were wide, particularly for the smaller active regular group. Additionally, identifying depression primarily through health records and ICD codes may miss subclinical or untreated depression, leading to outcome misclassification. Therefore, the findings regarding depression should be interpreted cautiously as comparable inverse associations rather than definitive evidence of superiority for any specific activity pattern. Finally, given the observational design, residual and unmeasured confounding remains a notable concern. Although our findings remained robust after adjusting for a comprehensive set of sociodemographic and lifestyle covariates—including additional sensitivity analyses for sleep, sedentary time, overall health status, and gastrointestinal medication use—we lacked sufficiently granular data to account for several other important unmeasured confounders. These include specific baseline comorbidities, baseline anxiety severity, prior mental health status, and detailed healthcare utilization behaviors. These unmeasured factors may independently influence both physical activity levels and the diagnosis of IBS or depression. Furthermore, causal relationships cannot be established from this observational design, and future randomized controlled trials are required to confirm whether the observed associations reflect true causal benefits. Nonetheless, to partially mitigate these biases, we excluded participants with incident outcomes in the previous 2 years after accelerometry or those diagnosed with major potentially confounding conditions.

## Conclusion

This large-scale, accelerometer-based study demonstrates a nonlinear inverse association between MVPA and IBS risk, with a minimal candidate dose of ~80 min/week and optimal risk reduction around 225 min/week. Both the WW and regularly active patterns were similarly associated with a lower IBS risk, and higher MVPA was associated with lower depression risk among IBS patients, with both the WW and regular patterns demonstrating comparable inverse associations. These findings highlight that flexible PA approaches, including concentrated weekend activity, represent potential practical approaches for the risk reduction and psychological management of IBS. Future research should further explore dose–response relationships of objectively measured PA across diverse health outcomes to define evidence-based activity thresholds for different populations.

## Data Availability

The datasets presented in this article are not readily available because the UK Biobank data are not publicly available due to participant confidentiality and ethical restrictions. Researchers can apply for access through the UK Biobank Access Management System at https://www.ukbiobank.ac.uk/enable-your-research/apply-for-access.
