# Supplementary material for: Accelerometer-derived “weekend warrior” and regular physical activity on long-term risk of irritable bowel syndrome and subsequent depression: a large-scale prospective cohort study
Source: Front Public Health. 2026 Jun 30;14:1865206. doi: 10.3389/fpubh.2026.1865206 (PMC13364927; doi:10.3389/fpubh.2026.1865206)
Supplement: Supplementary file 1 [file Data_Sheet_1.docx]

**Supplementary materials**

**Accelerometer-Derived “Weekend Warrior” and Regular Physical Activity on Long-term Risk of Irritable Bowel Syndrome and Subsequent Depression: A Large-scale Prospective Cohort Study**

**Chujie Tu^1#^, Xin Yang^2#^, Yan Wang^3#^, Jiaqi Hu^1^, Xiaohua Hou^1^, Tao Bai^4,5,6*^ and Li Liu^3,7,8,*^**

^1^ Division of Gastroenterology, Union Hospital, Tongji Medical College, Huazhong University of Science and Technology, Wuhan, Hubei, 430022, P.R. China.

^2^ Department of Endoscopy, Zhejiang Cancer Hospital, Hangzhou Institute of Medicine (HIM), Chinese Academy of Sciences, Hangzhou, Zhejiang 310022, China.

^3^ Department of Epidemiology and Biostatistics, Ministry of Education Key Lab of Environment and Health, School of Public Health, Tongji Medical College, Huazhong University of Science and Technology, Wuhan, Hubei, 430030, P.R. China.

^4^ Department of Gastroenterology, Tianyou Hospital, College of Medicine, Wuhan University of Science and Technology, Wuhan 430065, PR China.

^5^ Department of Clinical Medicine, College of Medicine, Wuhan University of Science and Technology, Wuhan 430065, PR China.

^6^ Division of Gastroenterology, The Central Hospital of Wuhan, Tongji Medical College, Huazhong University of Science and Technology, Wuhan, China

^7^ Hubei Provincial Clinical Research Center for Colorectal Cancer, Wuhan, Hubei, 430000, P.R. China.

^8^ Wuhan Clinical Research Center for Colorectal Cancer, Wuhan, Hubei, 430000, P.R. China.

#Chujie Tu, Xin Yang and Yan Wang contributed equally to this work.

*** Correspondence:**

Li Liu and Tao Bai are co-corresponding authors for this work.

Li Liu (email: liul2012@hust.edu.cn) and Tao Bai (email: baitao@wust.edu.cn)

**Contents**

eMethod.(4)

Table S1(6)

Table S2(7)

Table S3(8)

Table S4(9)

Table S5(10)

Table S6(11)

Table S7(12)

Table S8(13)

Figure S1(14)

Figure S2(15)

Figure S3(16)

Figure S4(17)

Figure S5(18)

Figure S6(19)

Figure S7(20)

Figure S8(21)

Figure S9(22)

Reference(23)

**eMethod.** Details about covariates assessment.

Age, sex and race were obtained at baseline. Body mass index (BMI) was calculated as weight divided by height squared (kg/m²) during the initial Assessment Centre visit. Smoking status, alcohol intake, diet quality, educational attainment, employment status, Townsend Deprivation Index (TDI), family history of depression were obtained from the Touchscreen questionnaire.

The race was classified as white and non-white. Body mass index (BMI) was calculated as weight (kilograms) divided by the square of height (meters), and classified as normal (<25.0), overweight (25.0-29.9) and obese (≥30.0). Smoking status was classified as never, current, or former smoker. Alcohol grams were calculated based on alcohol intake frequency and quantity questionnaire according to previous studies (1). Alcohol intake was classified as never or moderate (<28 grams/day for men, and <14 grams/day for women), or heavy drinking (≥28 grams/day for men, and ≥14 grams/day for women). Educational attainment was converted from categorical qualifications to equivalent years of schooling following a standardised mapping protocol previously adopted in UK Biobank studies(2,3): College/University degree = 20; NVQ/HND/HNC = 19; other professional qualifications = 15; A/AS levels = 13; O levels/GCSEs = 10; CSEs = 10; and none of the above = 7 years. For stratified analyses, educational attainment was further classified into three categories: high (college degree or above), medium (high school or equivalent), and low (less than high school). Diet quality was assessed using a previously validated UK Biobank dietary index incorporating seven components: vegetables (≥4 tablespoons/day), fruit (≥3 servings/day), fish (≥2 servings/week), processed meat (≤2 servings/week), unprocessed red meat (≤5 servings/week), whole grains (≥3 servings/day), and refined grains (≤1.5 servings/day). One point was assigned for meeting each ideal intake criterion (4). Based on the total score, participants meeting ≥4, 2–3, or ≤1 of these criteria were classified as having good, normal, or poor diet quality, respectively. TDI is a simple census-based index of material deprivation calculated by the combination of 4 census variables, including households without a car, overcrowded households, household not owner-occupied, and persons unemployed. TDI was classified as low, medium and high by quartiles.

**Table S1.** Baseline characteristics of UK Biobank participants with and without accelerometer data.

|  | **UKB Total** | **Accelerometer Subset** |  |
| --- | --- | --- | --- |
| **Characteristic** | N = 502,401 | N = 103,660 | **SMD** |
| Age at recruitment (years) | 56.53 (8.10) | 56.06 (7.84) | 0.059 |
| Sex |  |  | 0.036 |
| Female | 273320 (54.4) | 58267 (56.2) |  |
| Male | 229081 (45.6) | 45393 (43.8) |  |
| Race/ethnicity |  |  | **0.108** |
| Non-white | 27020 (5.4) | 3315 (3.2) |  |
| White | 472603 (94.6) | 99978 (96.8) |  |
| Body mass index |  |  | **0.148** |
| Normal (<25.0) | 164891 (33.0) | 40543 (39.2) |  |
| Overweight (25.0-29.9) | 212103 (42.5) | 42600 (41.2) |  |
| Obese (≥30.0) | 122340 (24.5) | 20289 (19.6) |  |
| Smoking status |  |  | **0.130** |
| Current | 52602 (10.5) | 7105 (6.9) |  |
| Former | 173184 (34.7) | 37257 (36.0) |  |
| Never | 273663 (54.8) | 59024 (57.1) |  |
| Alcohol intake (g/week) | 124.09 (144.55) | 127.91 (136.33) | 0.027 |
| Diet quality |  |  | 0.080 |
| Good | 76987 (15.4) | 18137 (17.5) |  |
| Normal | 281463 (56.2) | 59207 (57.2) |  |
| Poor | 142018 (28.4) | 26215 (25.3) |  |
| Townsend Deprivation Index | -1.29 (3.09) | -1.71 (2.83) | **0.140** |
| Employment status |  |  | 0.080 |
| Employed | 283376 (56.7) | 62744 (60.7) |  |
| Unemployed | 216182 (43.3) | 40694 (39.3) |  |
| Educational attainment (years) | 13.96 (4.97) | 15.37 (4.65) | **0.292** |

Data are presented as mean (standard deviation) for continuous variables and number (%) for categorical variables. SMD = standardized mean difference.

**Table S2.** Baseline characteristics of participants included versus excluded from the primary analysis within the accelerometer subset.

|  | **Primary Analysis** | **Excluded** |  |
| --- | --- | --- | --- |
| **Characteristic** | N = 84,799 | N = 18,861 | **SMD** |
| Age (years) | 61.81 (7.86) | 61.62 (7.91) | 0.024 |
| Sex |  |  | 0.094 |
| Female | 46947 (55.4) | 11320 (60.0) |  |
| Male | 37852 (44.6) | 7541 (40.0) |  |
| Race/ethnicity |  |  | 0.025 |
| Non-white | 2654 (3.1) | 661 (3.6) |  |
| White | 82145 (96.9) | 17833 (96.4) |  |
| Body mass index |  |  | 0.024 |
| Normal (<25.0) | 33237 (39.2) | 7306 (39.2) |  |
| Overweight (25.0-29.9) | 35059 (41.3) | 7541 (40.5) |  |
| Obese (≥30.0) | 16503 (19.5) | 3786 (20.3) |  |
| Smoking status |  |  | 0.032 |
| Current | 5712 (6.7) | 1393 (7.5) |  |
| Former | 30520 (36.0) | 6737 (36.2) |  |
| Never | 48567 (57.3) | 10457 (56.3) |  |
| Alcohol intake (g/week) | 129.13 (136.51) | 122.41 (135.39) | 0.049 |
| Diet quality |  |  | 0.016 |
| Good | 14911 (17.6) | 3226 (17.2) |  |
| Normal | 48520 (57.2) | 10687 (57.0) |  |
| Poor | 21368 (25.2) | 4847 (25.8) |  |
| Townsend Deprivation Index | -1.73 (2.82) | -1.62 (2.89) | 0.038 |
| Employment status |  |  | 0.007 |
| Employed | 51493 (60.7) | 11251 (60.4) |  |
| Unemployed | 33306 (39.3) | 7388 (39.6) |  |
| Educational attainment (years) | 15.39 (4.64) | 15.25 (4.65) | 0.030 |

Data are presented as mean (standard deviation) for continuous variables and number (%) for categorical variables. SMD = standardized mean difference.

**Table S3.** Baseline characteristics of participants in UK Biobank by depression in IBS patients in the secondary analysis.

| **Characteristic** | **Depression** | | | **P Value***^2^* |
| --- | --- | --- | --- | --- |
|  | **Overall** | **No** | **Yes** |  |
|  | N = 3,976*^1^* | N = 3,821*^1^* | N = 155*^1^* |  |
| **Age (years)** | 61.85 (7.70) | 61.88 (7.69) | 61.26 (7.86) | 0.271 |
| **Sex** |  |  |  | **0.048** |
| *Female* | 2,850 (71.7) | 2,728 (71.4) | 122 (78.7) |  |
| *Male* | 1,126 (28.3) | 1,093 (28.6) | 33 (21.3) |  |
| **Race/ethnicity** |  |  |  | >0.9 |
| *Non-white* | 81 (2.0) | 78 (2.0) | 3 (1.9) |  |
| *White* | 3,895 (98.0) | 3,743 (98.0) | 152 (98.1) |  |
| **Body mass index** |  |  |  | **0.022** |
| *Normal (<25.0)* | 1,754 (44.1) | 1,702 (44.5) | 52 (33.5) |  |
| *Overweight (25.0-29.9)* | 1,526 (38.4) | 1,453 (38.0) | 73 (47.1) |  |
| *Obese (≥30.0)* | 696 (17.5) | 666 (17.4) | 30 (19.4) |  |
| **Smoking status** |  |  |  | **0.007** |
| *Current* | 215 (5.4) | 199 (5.2) | 16 (10.3) |  |
| *Former* | 1,359 (34.2) | 1,300 (34.0) | 59 (38.1) |  |
| *Never* | 2,402 (60.4) | 2,322 (60.8) | 80 (51.6) |  |
| **Alcohol intake (g/week)** | 107.60 (116.32) | 107.72 (115.79) | 104.57 (129.10) | 0.214 |
| **Diet quality** |  |  |  | 0.535 |
| *Good* | 735 (18.5) | 707 (18.5) | 28 (18.1) |  |
| *Normal* | 2,286 (57.5) | 2,202 (57.6) | 84 (54.2) |  |
| *Poor* | 955 (24.0) | 912 (23.9) | 43 (27.7) |  |
| **Townsend Deprivation Index** | -1.91 (2.73) | -1.93 (2.73) | -1.47 (2.62) | **0.005** |
| **Employment status** |  |  |  | 0.234 |
| *Employed* | 2,313 (58.2) | 2,230 (58.4) | 83 (53.5) |  |
| *Unemployed* | 1,663 (41.8) | 1,591 (41.6) | 72 (46.5) |  |
| **Educational attainment (years)** | 15.10 (4.65) | 15.10 (4.64) | 14.92 (4.84) | 0.662 |
| **Family history of depression** | 676 (17.0) | 636 (16.6) | 40 (25.8) | **0.003** |
| *^1^* mean (SD) for continuous; n (%) for categorical | | | | |
| *^2^* Wilcoxon rank sum test; Pearson’s Chi-squared test; Fisher’s exact test | | | | |

**Table S4.** Associations between physical activity patterns with the incidence of IBS and depression in IBS patients, with weekend warriors as the reference group.

| **Physical activity pattern** | **Events/N** | **Model 1** | **Model 2** |
| --- | --- | --- | --- |
| **Incidence of IBS** |  |  |  |
| Active Weekend Warrior | 350/36336 | 1 (ref) | 1 (ref) |
| Inactive | 422/28028 | 1.43 (1.24,1.65) | 1.33 (1.14,1.54) |
| Active Regular | 190/20435 | 0.97 (0.82,1.16) | 0.97 (0.81,1.16) |
| **Depression in IBS** |  |  |  |
| Active Weekend Warrior | 45/1583 | 1 (ref) | 1 (ref) |
| Inactive | 84/1584 | 1.85 (1.28,2.66) | 1.65 (1.13,2.40) |
| Active Regular | 26/809 | 1.10 (0.68,1.79) | 1.07 (0.66,1.74) |

Analyses were adjusted for age (continuous), sex, race (white or non-white), smoking status (never, former or current), alcohol grams (continuous), educational attainment (continuous), BMI (<25.0, 25.0–29.9 or ≥30.0 kg/m²), Townsend deprivation index (continuous), employment status (yes or no), and diet quality (good, normal or poor). For subsequent depression analyses, family history of depression (yes or no) was additionally adjusted. Weekend warriors served as the reference group.

**Table S5.** Associations between physical activity duration and patterns with the incidence of IBS and depression in IBS patients in Model 2 with additional adjustment for sleep duration.

|  | **Incidence of IBS** | | **Depression in IBS** | |
| --- | --- | --- | --- | --- |
|  | Events/N | HR (95% CI) | Events/N | HR (95% CI) |
| **Physical activity duration** |  |  |  |  |
| Quartile 1 | 344/21200 | 1 (ref) | 60/994 | 1 (ref) |
| Quartile 2 | 230/21200 | 0.73 (0.62,0.86) | 38/994 | 0.70 (0.46,1.06) |
| Quartile 3 | 219/21200 | 0.75 (0.63,0.89) | 29/994 | 0.54 (0.34,0.85) |
| Quartile 4 | 169/21199 | 0.64 (0.53,0.78) | 28/994 | 0.55 (0.34,0.89) |
| p for trend |  | <0.001 |  | 0.005 |
| **Physical activity pattern** |  |  |  |  |
| Inactive | 422/28028 | 1 (ref) | 84/1584 | 1 (ref) |
| Active Regular | 190/20435 | 0.74 (0.62,0.89) | 26/809 | 0.66 (0.41,1.04) |
| Active Weekend Warrior | 350/36336 | 0.76 (0.66,0.88) | 45/1583 | 0.61 (0.42,0.89) |

Analyses were adjusted for age (continuous), sex, race (white or non-white), smoking status (never, former or current), alcohol grams (continuous), educational attainment (continuous), BMI (<25.0, 25.0-29.9 or ≥30.0 kg/m^2^), Townsend deprivation index (continuous), employment status (yes or no), diet quality (good, normal or poor), and sleep duration (continuous). For subsequent depression analyses, family history of depression (yes or no) was additionally adjusted.

**Table S6.** Associations between physical activity duration and patterns with the incidence of IBS and depression in IBS patients in Model 2 with additional adjustment for sedentary behavior.

|  | **Incidence of IBS** | | **Depression in IBS** | |
| --- | --- | --- | --- | --- |
|  | Events/N | HR (95% CI) | Events/N | HR (95% CI) |
| **Physical activity duration** |  |  |  |  |
| Quartile 1 | 344/21200 | 1 (ref) | 60/994 | 1 (ref) |
| Quartile 2 | 230/21200 | 0.73 (0.62,0.86) | 38/994 | 0.70 (0.46,1.06) |
| Quartile 3 | 219/21200 | 0.75 (0.63,0.89) | 29/994 | 0.54 (0.34,0.85) |
| Quartile 4 | 169/21199 | 0.64 (0.53,0.79) | 28/994 | 0.55 (0.34,0.90) |
| p for trend |  | <0.001 |  | 0.006 |
| **Physical activity pattern** |  |  |  |  |
| Inactive | 422/28028 | 1 (ref) | 84/1584 | 1 (ref) |
| Active Regular | 190/20435 | 0.75 (0.62,0.89) | 26/809 | 0.66 (0.41,1.04) |
| Active Weekend Warrior | 350/36336 | 0.76 (0.66,0.89) | 45/1583 | 0.61 (0.42,0.89) |

Analyses were adjusted for age (continuous), sex, race (white or non-white), smoking status (never, former or current), alcohol grams (continuous), educational attainment (continuous), BMI (<25.0, 25.0-29.9 or ≥30.0 kg/m^2^), Townsend deprivation index (continuous), employment status (yes or no), diet quality (good, normal or poor), and sedentary behavior (continuous). For subsequent depression analyses, family history of depression (yes or no) was additionally adjusted.

**Table S7.** Associations between physical activity duration and patterns with the incidence of IBS and depression in IBS patients in Model 2 with additional adjustment for gastrointestinal medication use.

|  | **Incidence of IBS** | | **Depression in IBS** | |
| --- | --- | --- | --- | --- |
|  | Events/N | HR (95% CI) | Events/N | HR (95% CI) |
| **Physical activity duration** |  |  |  |  |
| Quartile 1 | 344/21200 | 1 (ref) | 60/994 | 1 (ref) |
| Quartile 2 | 230/21200 | 0.74 (0.63,0.88) | 38/994 | 0.71 (0.47,1.08) |
| Quartile 3 | 219/21200 | 0.78 (0.65,0.93) | 29/994 | 0.55 (0.35,0.87) |
| Quartile 4 | 169/21199 | 0.67 (0.55,0.82) | 28/994 | 0.57 (0.35,0.91) |
| p for trend |  | <0.001 |  | 0.007 |
| **Physical activity pattern** |  |  |  |  |
| Inactive | 422/28028 | 1 (ref) | 84/1584 | 1 (ref) |
| Active Regular | 190/20435 | 0.77 (0.64,0.92) | 26/809 | 0.66 (0.42,1.05) |
| Active Weekend Warrior | 350/36336 | 0.78 (0.67,0.91) | 45/1583 | 0.62 (0.43,0.91) |

Analyses were adjusted for age (continuous), sex, race (white or non-white), smoking status (never, former or current), alcohol grams (continuous), educational attainment (continuous), BMI (<25.0, 25.0-29.9 or ≥30.0 kg/m^2^), Townsend deprivation index (continuous), employment status (yes or no), diet quality (good, normal or poor), and gastrointestinal medication use (yes or no). For subsequent depression analyses, family history of depression (yes or no) was additionally adjusted.

**Table S8.** Associations between physical activity duration and patterns with the incidence of IBS and depression in IBS patients in Model 2 with additional adjustment for general health.

|  | **Incidence of IBS** | | **Depression in IBS** | |
| --- | --- | --- | --- | --- |
|  | Events/N | HR (95% CI) | Events/N | HR (95% CI) |
| **Physical activity duration** |  |  |  |  |
| Quartile 1 | 344/21143 | 1 (ref) | 60/990 | 1 (ref) |
| Quartile 2 | 229/21166 | 0.76 (0.64,0.90) | 38/992 | 0.74 (0.49,1.12) |
| Quartile 3 | 218/21169 | 0.79 (0.67,0.95) | 29/993 | 0.58 (0.37,0.92) |
| Quartile 4 | 169/21174 | 0.69 (0.57,0.85) | 28/994 | 0.62 (0.38,1.01) |
| p for trend |  | <0.001 |  | 0.020 |
| **Physical activity pattern** |  |  |  |  |
| Inactive | 421/27957 | 1 (ref) | 84/1579 | 1 (ref) |
| Active Regular | 189/20403 | 0.78 (0.65,0.94) | 26/808 | 0.70 (0.44,1.11) |
| Active Weekend Warrior | 350/36292 | 0.81 (0.70,0.94) | 45/1582 | 0.65 (0.45,0.95) |

Analyses were adjusted for age (continuous), sex, race (white or non-white), smoking status (never, former or current), alcohol grams (continuous), educational attainment (continuous), BMI (<25.0, 25.0-29.9 or ≥30.0 kg/m^2^), Townsend deprivation index (continuous), employment status (yes or no), diet quality (good, normal or poor), and general health (better or poorer). For subsequent depression analyses, family history of depression (yes or no) was additionally adjusted.


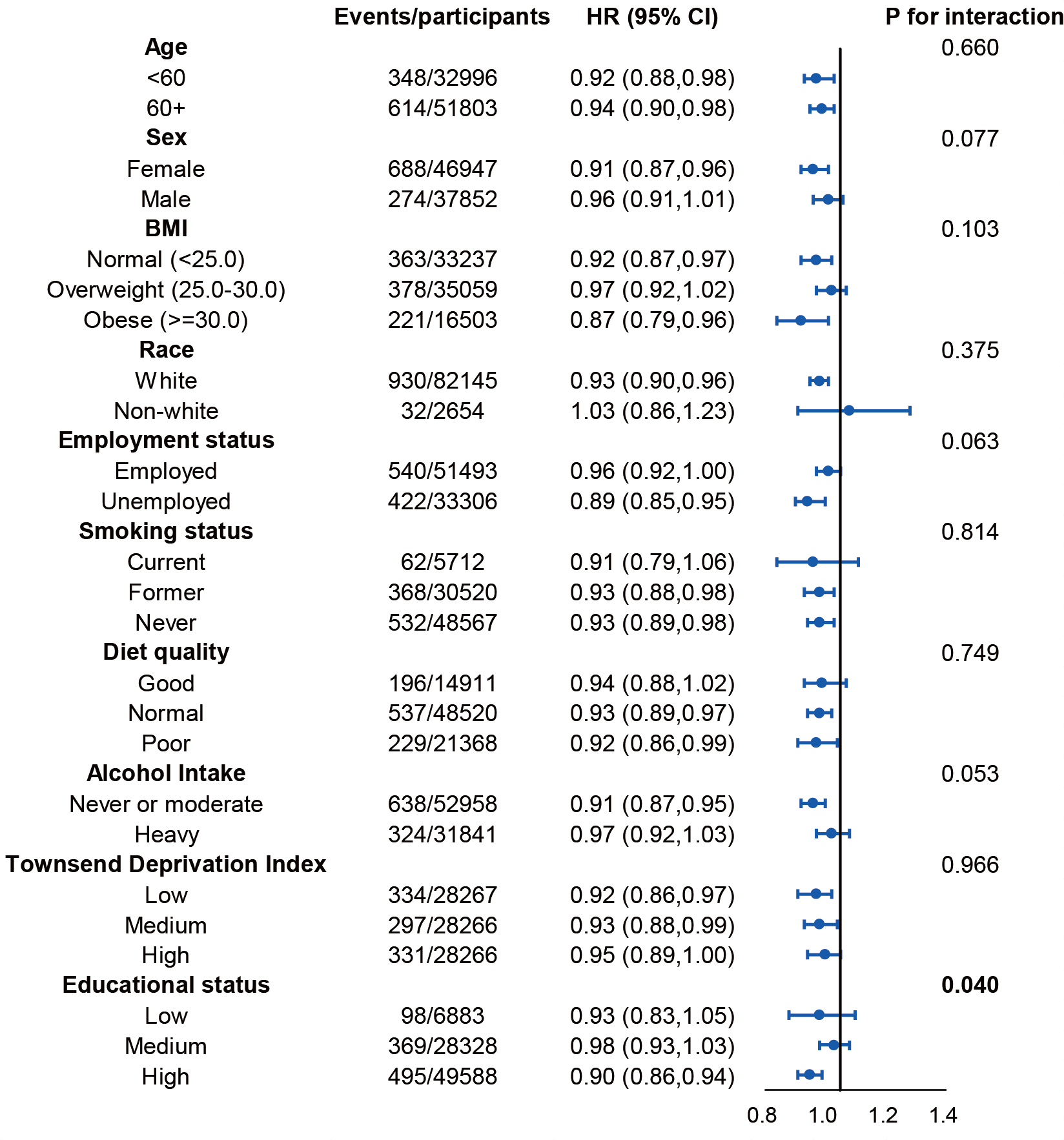


**Figure S1. Stratified analysis of the association between physical activity duration and the incidence of IBS.**

Analyses were adjusted for age (continuous), sex, race (white or non-white), smoking status (never, former or current), alcohol grams (continuous), educational status (high, medium, low), BMI (<25.0, 25.0-29.9 or ≥30.0 kg/m^2^), Townsend deprivation index (continuous), employment status (yes or no), diet quality (good, normal or poor).


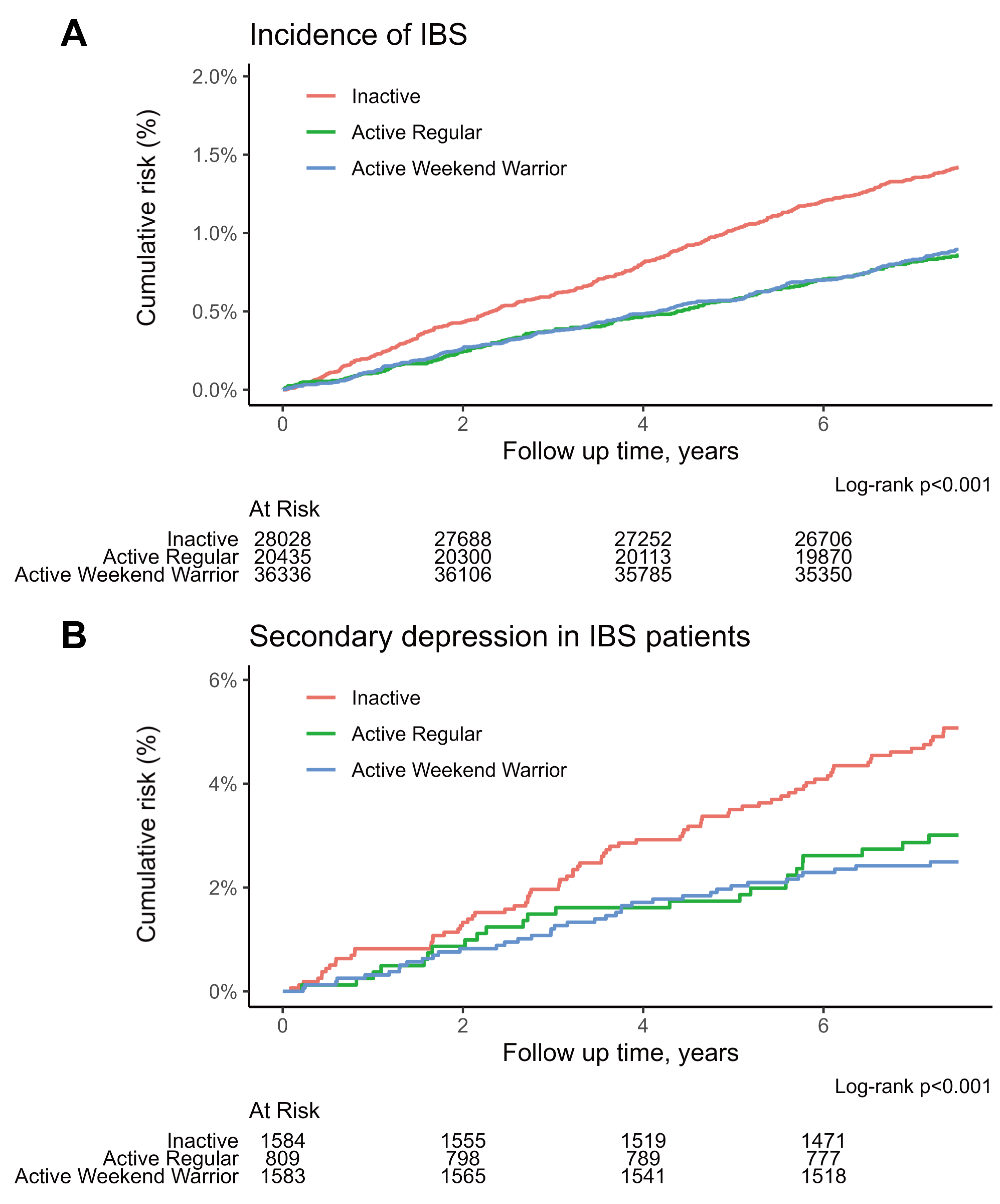


**Figure S2. Cumulative risk of the incidence of IBS and depression in IBS patients stratified by physical activity patterns.**

(A) Incidence of IBS; (B) Secondary depression in IBS patients. Plots depict the crude cumulative risk of the disease and the number at-risk over time is depicted below each plot.


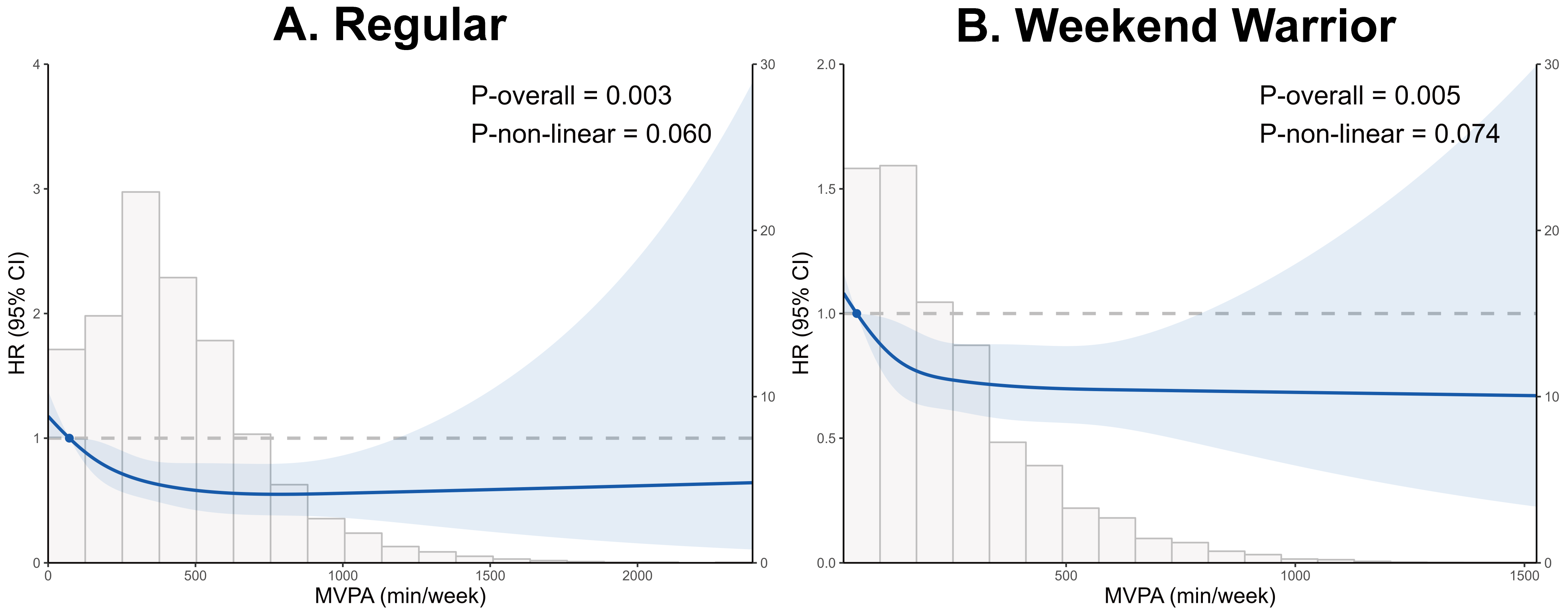


**Figure S3.** **Association of physical activity duration (min/week) with the risk of incident IBS stratified by physical activity patterns.**

Hazard ratios are indicated by solid lines and 95% CIs by shaded areas. Histogram shows the distribution of physical activity duration. Results were adjusted for age (continuous), sex, race (white or non-white), smoking status (never, former or current), alcohol grams (continuous), educational attainment (continuous), BMI (<25.0, 25.0-29.9 or ≥30.0 kg/m^2^), Townsend deprivation index (continuous), employment status (yes or no), diet quality (good, normal or poor).


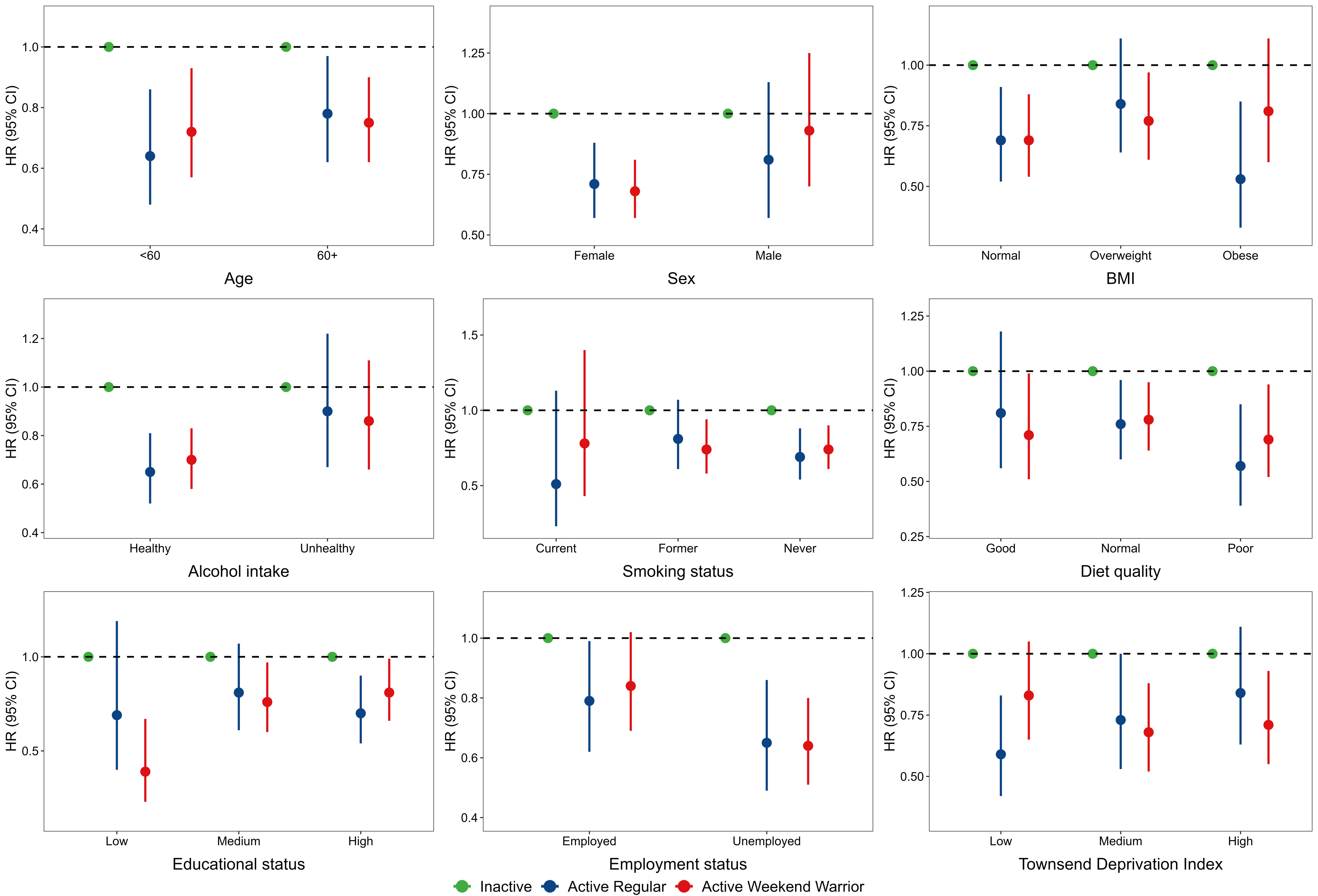


**Figure S4. Stratified analysis of the association between physical activity patterns and the incidence of IBS.**

Analyses were adjusted for age (continuous), sex, race (white or non-white), smoking status (never, former or current), alcohol grams (continuous), educational status (high, medium, low), BMI (<25.0, 25.0-29.9 or ≥30.0 kg/m^2^), Townsend deprivation index (continuous), employment status (yes or no), diet quality (good, normal or poor).


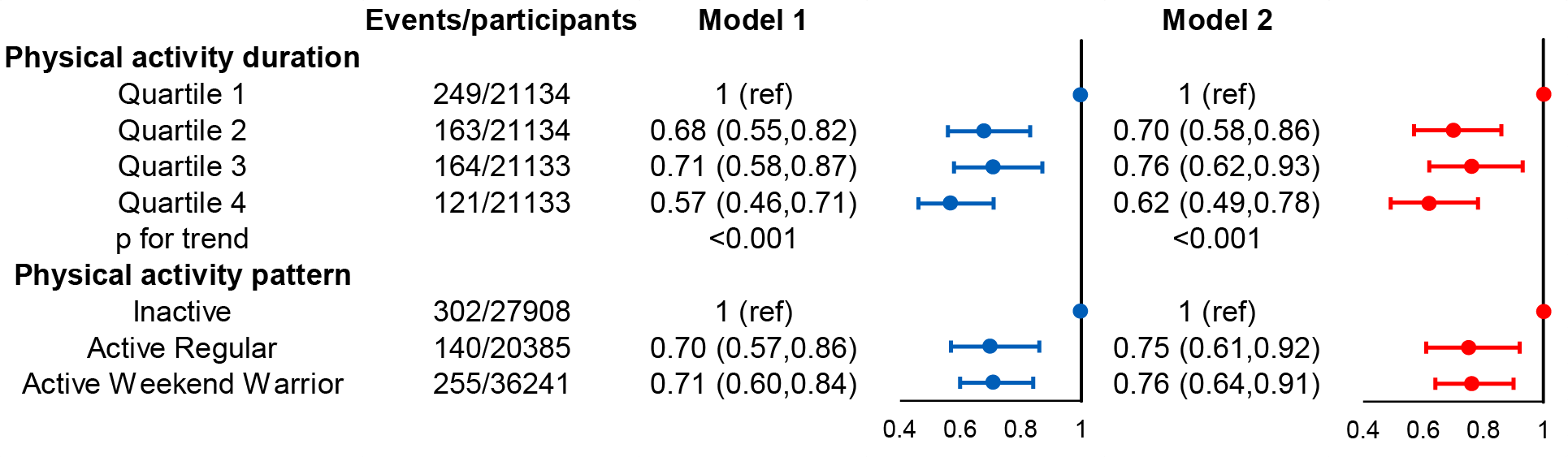


**Figure S5.** **Association between physical activity duration and patterns with the incidence of IBS with a 2-year blanking period following accelerometer measurement.**

Model 1 was adjusted for age (continuous), sex. Model 2 was further adjusted for race (white or non-white), smoking status (never, former or current), alcohol grams (continuous), educational attainment (continuous), BMI (<25.0, 25.0-29.9 or ≥30.0 kg/m^2^), Townsend deprivation index (continuous), employment status (yes or no), diet quality (good, normal or poor).


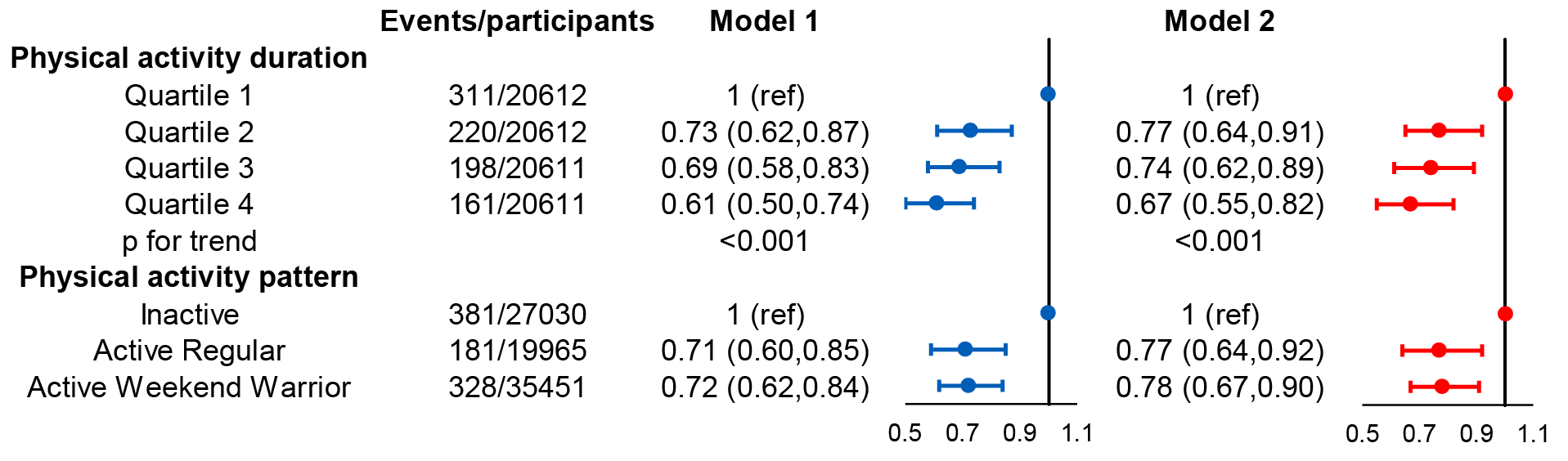


**Figure S6.** **Association between physical activity duration and patterns with the incidence of IBS further excluding individuals with prior diagnoses of noninfective gastroenteritis, colitis, or other pancreatic disorders.**

Model 1 was adjusted for age (continuous), sex. Model 2 was further adjusted for race (white or non-white), smoking status (never, former or current), alcohol grams (continuous), educational attainment (continuous), BMI (<25.0, 25.0-29.9 or ≥30.0 kg/m^2^), Townsend deprivation index (continuous), employment status (yes or no), diet quality (good, normal or poor).


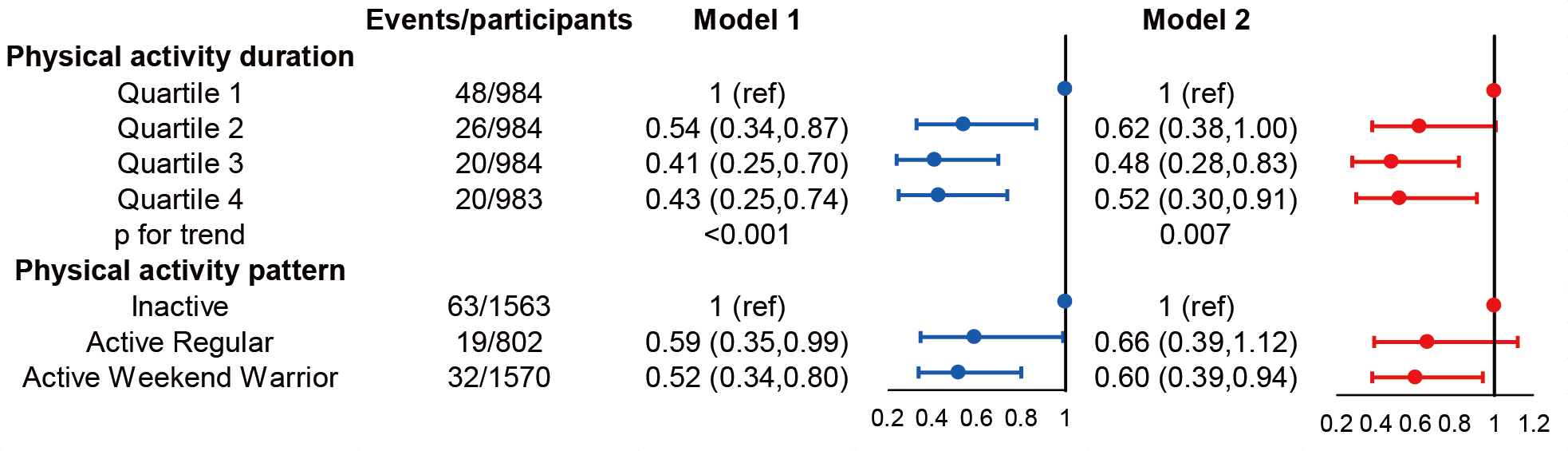


**Figure S7. Association between physical activity duration and patterns with depression in IBS patients with a 2-year blanking period following accelerometer measurement.**

Model 1 was adjusted for age (continuous), sex. Model 2 was further adjusted for race (white or non-white), smoking status (never, former or current), alcohol grams (continuous), educational attainment (continuous), BMI (<25.0, 25.0-29.9 or ≥30.0 kg/m^2^), Townsend Deprivation Index (continuous), employment status (yes or no), diet quality (good, normal or poor), family history of depression (yes or no).


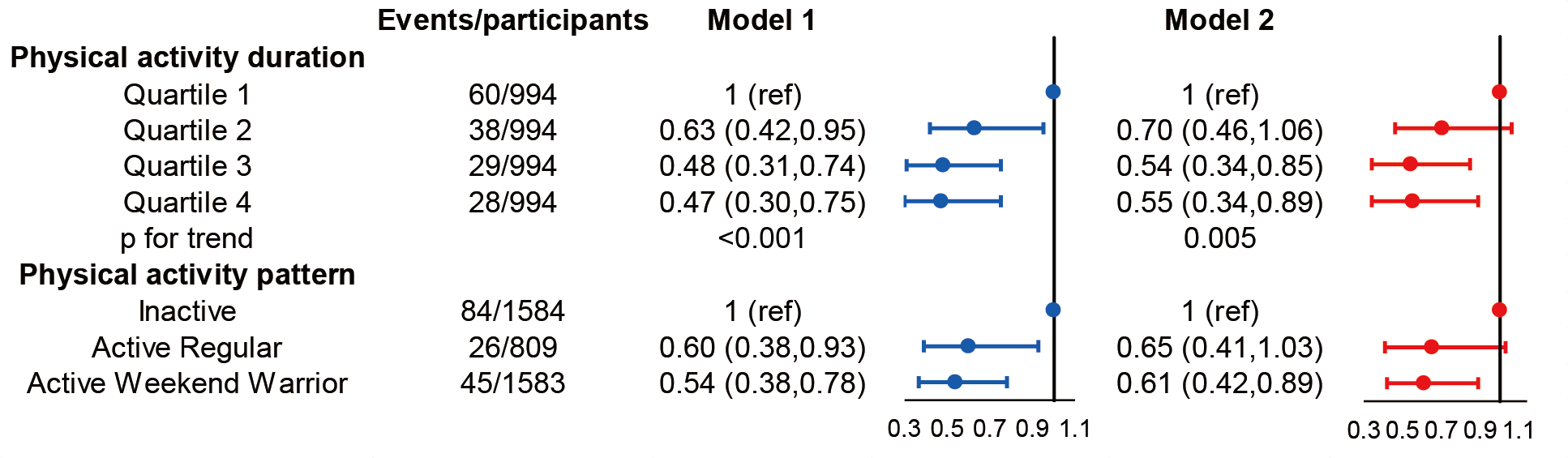


**Figure S8.** **Association between physical activity duration and patterns with depression in IBS patients further excluding individuals with prior diagnoses of noninfective gastroenteritis, colitis, or other pancreatic disorders.**

Model 1 was adjusted for age (continuous), sex. Model 2 was further adjusted for race (white or non-white), smoking status (never, former or current), alcohol grams (continuous), educational attainment (continuous), BMI (<25.0, 25.0-29.9 or ≥30.0 kg/m^2^), Townsend Deprivation Index (continuous), employment status (yes or no), diet quality (good, normal or poor), family history of depression (yes or no).


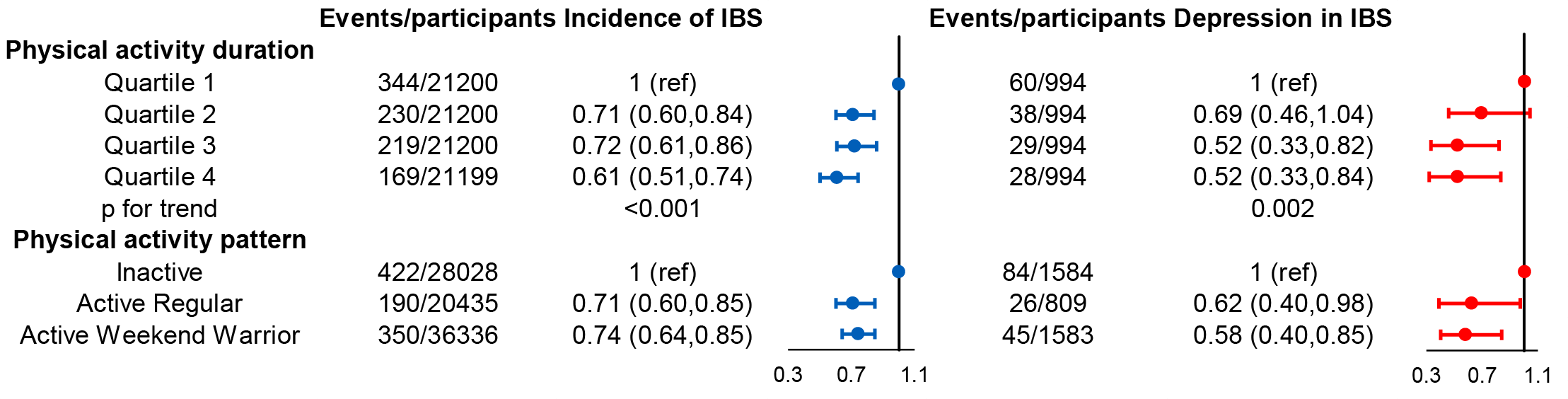


**Figure S9.** **Associations between physical activity duration and patterns with the incidence of IBS and depression in IBS patients in Model 2 with the removal of Body Mass Index (BMI).**

Analyses were adjusted for age (continuous), sex, race (white or non-white), smoking status (never, former or current), alcohol grams (continuous), educational attainment (continuous), Townsend deprivation index (continuous), employment status (yes or no), diet quality (good, normal or poor). For subsequent depression analyses, family history of depression (yes or no) was additionally adjusted.

**Reference**

1. Halford JL, Weng L-C, Choi SH, Jurgens SJ, Morrill VN, Khurshid S, Trinquart L, Benjamin EJ, Ellinor PT, Lubitz SA. Associations between alcohol intake and genetic predisposition with atrial fibrillation risk in a national biobank. *Circ Genom Precis Med* (2020) 13:e003111. doi: 10.1161/CIRCGEN.120.003111

2. Okbay A, Wu Y, Wang N, Jayashankar H, Bennett M, Nehzati SM, Sidorenko J, Kweon H, Goldman G, Gjorgjieva T, et al. Polygenic prediction of educational attainment within and between families from genome-wide association analyses in 3 million individuals. *Nat Genet* (2022) 54:437–449. doi: 10.1038/s41588-022-01016-z

3. Khurshid S, Al-Alusi MA, Churchill TW, Guseh JS, Ellinor PT. Accelerometer-derived “weekend warrior” physical activity and incident cardiovascular disease. *JAMA* (2023) 330:247. doi: 10.1001/jama.2023.10875

4. Bhattacharya R, Zekavat SM, Uddin MM, Pirruccello J, Niroula A, Gibson C, Griffin GK, Libby P, Ebert BL, Bick A, et al. Association of diet quality with prevalence of clonal hematopoiesis and adverse cardiovascular events. *JAMA Cardiol* (2021) 6:1069–1077. doi: 10.1001/jamacardio.2021.1678
